# Association between Single-Nucleotide Polymorphism in MicroRNA Target Site of *DDB2* and Risk of Hepatocellular Carcinoma in a Southern Chinese Population

**DOI:** 10.1155/2020/8528747

**Published:** 2020-02-08

**Authors:** Moqin Qiu, Yingchun Liu, Zihan Zhou, Yanji Jiang, Qiuling Lin, Rongrui Huo, Xiumei Liang, Xiangyuan Yu, Xianguo Zhou, Hongping Yu

**Affiliations:** ^1^Guangxi Medical University Cancer Hospital, Nanning 530021, China; ^2^School of Public Health, Guangxi Medical University, Nanning 530021, China; ^3^Affiliated Hospital of Guilin Medical University, Guilin 541004, China; ^4^School of Public Health, Guilin Medical University, Guilin 541004, China

## Abstract

Damage-specific DNA-binding protein 2 (DDB2) is a DNA repair protein mainly involved in nucleotide excision repair, which plays a pivotal role in maintaining genomic stability. In this study, we evaluated the association of single-nucleotide polymorphism (SNP) rs1050244 in miRNA target site of *DDB2* gene with risk of hepatocellular carcinoma (HCC) among 1073 HCC patients and 1119 cancer-free controls in a southern Chinese population. Our results showed that no statistically significant association was found between *DDB2* rs1050244 and HCC risk. In further analysis stratified by age, sex, smoking, alcohol drinking, and HBV infection status, we found that individuals carrying the CT/TT genotypes of SNP rs1050244 had a significantly decreased risk of HCC compared with those with the CC genotype among non-HBV infected population (adjusted OR = 0.31, 95% CI = 0.13–0.72), and a significant interaction was found between this SNP and HBV infection (*P*_interaction_=0.002). Our results suggested that the *DDB2* rs1050244 C>T polymorphism was associated with the decreased risk of HCC among non-HBV infected population. Further studies with larger sample sizes are needed to validate our findings.

## 1. Introduction

Liver cancer is one of the most common cancers worldwide, with an estimated 841,000 new cases and 782,000 deaths reported in 2018 [1]. Hepatocellular carcinoma (HCC) is the most common histological type, accounting for approximately 75–85% of all primary liver cancer patients [2]. Hepatitis B/C virus (HBV/HCV) infection, Aflatoxin exposure, alcohol drinking, and smoking are the main risk factors for HCC [3]. Chronic HBV infection is the most important risk factor for HCC in China [4]. However, only a small part of HBV carriers eventually develop HCC in their lifetime, suggesting that individual genetic susceptibility contributes to HCC [5, 6].

Nucleotide excision repair (NER) is an important versatile DNA repair mechanism that plays a critical role in preventing carcinogenesis [7, 8]. Damage-specific DNA-binding protein 2 (*DDB2*) gene, located on chromosome 11p11.2, is the major component of the NER pathway. *DDB2* gene encodes a protein that is a subunit of the damaged DNA-binding protein (DDB), which participates in the recognition of DNA damage and the initiation of NER process [9]. DDB2 has been reported to be abnormal expressed in several cancers [10, 11]. DDB2 was downregulated in high-grade colon cancer, and low expression of DDB2 inhibited epithelial-to-mesenchymal transition (EMT) of the colon cancer cells, while upregulated the expression of DDB2 inhibited metastasis [12]. Moreover, overexpression of DDB2 could inhibit the invasion and migration of breast cancer [13].

Single-nucleotide polymorphisms (SNPs) are a type of common genetic variations in the human genome. MicroRNAs (miRNAs) are a class of noncoding small RNAs that lead to cleavage of target mRNA or repression of mRNA translation by binding to target sites in the 3′ untranslated region (3′ UTR) of mRNAs [14]. It has been demonstrated that SNPs residing in noncoding regions could affect transcriptional regulation or posttranscriptional gene expression and thus contribute to the susceptibility to cancers [15, 16]. In this study, we hypothesized that SNPs in miRNA-binding site of *DDB2* may contribute to the susceptibility to HCC. To test this hypothesis, we conducted a case-control study of 1073 HCC cases and 1119 cancer-free controls in a southern Chinese population.

## 2. Materials and Methods

### 2.1. Study Population

A total of 1073 patients with newly diagnosed HCC and 1119 cancer-free controls were recruited in the First Affiliated Hospital of Guangxi Medical University, Guangxi Medical University Cancer hospital, and the First Affiliated Hospital of Guilin Medical University from August 2007 to November 2011. HCC patients with second primary tumors, metastasized cancers, or the history of previous radiotherapy or chemotherapy before the recruitment were excluded. All participants were genetically unrelated. Having signed a written informed consent during the interview, all subjects enrolled in the study were interviewed to collect demographic information and history of smoking and alcohol drinking. Of the 1073 HCC patients, 563 HCC patients had relatively complete clinical information, such as AFP level, tumor size, tumor number, BCLC stage, cancer embolus, and cirrhosis. 3–5 mL peripheral blood sample was obtained from each study object, of which 1 ml was used to detect HBV infection status. The research protocol was approved by Ethical Committee Review Board of Guilin Medical University.

### 2.2. Selection and Genotyping of the miRNA-Binding Site SNPs

The *DDB2* gene 3′ UTR SNPs were screened and identified by NIEHS SNPinfo (http://snpinfo.niehs.nih.gov/). The selection criteria are as follows: ① SNPs were located in the 3′ UTR region of *DDB2* gene and were potential miRNA target sites. ② Minor allele frequency (MAF) in Chinese Han population ≥0.05. ③The SNP linkage disequilibrium is less than 0.8. Finally, rs1050244 (C>T) of *DDB2* gene was selected for the study.

Genomic DNA was extracted from peripheral blood by phenol-chloroform extraction and stored at −80°C. The Agena MassARRAY SNP genotyping system (Agena; San Diego, CA) was used for genotyping following the manufacturer's instructions. The primers used for *DDB2* of rs1050244 were: F: 5′-ACGTTGGATGCCAACCCTAACCTTGGATAC-3′ and R: 5′-ACGTTGGATGACACATGGGATCAAGTCCTG-3′. The results of genotyping were analyzed with the MassARRAY Typer software version 4.0. To ensure quality control, randomly 10% of the samples were selected for repeating the genotyping assays, and the reproducibility was 100%.

### 2.3. Statistical Analysis

Distribution differences of demographic characteristics between cases and controls were assessed by *χ*^2^ test. Hardy–Weinberg equilibrium (HWE) in controls was tested by a goodness of fit *χ*^2^ test. Odds ratios (ORs) and 95% confidence intervals (CIs) were calculated for the associations between *DDB2* rs1050244 and the risk of HCC by multivariate logistic regression model. Associations between the genotypes and the risk of HCC among subgroups of age, sex, smoking, or drinking status and HBV infection status were evaluated by stratification analysis. All statistical tests were two-sided with a 0.05 significance level and were conducted using SPSS 18.0 software (SPSS, Chicago, IL, USA).

## 3. Results

### 3.1. Characteristics of the Study Population

As shown in Table 1, there were no statistical differences in the distributions of age and sex between the HCC patients and controls subjects (*P*=0.172 and 0.338, respectively). However, there were more smokers (41.8% versus 22.6%), drinkers (39% vs. 20.1%), and HBV-infected individuals (83.8% vs. 10.2%) in the cases than that in the controls.

### 3.2. Associations between *DDB2* rs1050244 Genotypes and HCC Risk

Table 2 summarizes the distributions of the genotype frequencies for *DDB2* rs1050244 and their associations with the risk of HCC. The genotype frequencies of rs1050244 among the controls were in agreement with the Hardy–Weinberg equilibrium (*P*=0.57). The frequencies of rs105024 CC, CT, and TT genotypes were 92.26%, 17.46%, and 0.28% in HCC cases, while they were 89.9%, 9.74%, and 0.36% in the controls, respectively. However, the Chi-squared test revealed that no significant differences in the genotype distributions were found between the cases and controls (*P* > 0.05). We also investigated the associations between *DDB2* rs1050244 and the clinical features of 563 HCC patients, such as AFP level, tumor size, tumor number, BCLC stage, cancer embolus, and cirrhosis, but failed to find any significant association ([Supplementary-material supplementary-material-1]).

We further conducted the stratified analysis on the association between *DDB2* rs1050244 and HCC risk by age, sex, smoking status, alcohol drinking status, and HBV infection status. As shown in Table 3, compared with the CC genotype, the CT/TT genotypes of rs1050244 were significantly associated with the decreased risk of HCC among non-HBV-infected population (adjusted OR = 0.31, 95% CI = 0.13–0.72, *P*=0.006), and a significant interaction between this polymorphism and HBV infection (*P*_interaction_=0.002) was observed. However, no significant associations were found between *DDB2* rs1050244 and HCC risk in the stratification analyses by age, sex, smoking, and alcohol drinking status.

## 4. Discussion

In the current case-control study, we investigated the association between the *DDB2* rs1050244 polymorphism and the risk of HCC in a southern Chinese population. Although the main effect of this polymorphism on the risk of HCC was not detected, we found that the rs1050244 CT/TT genotypes decreased the risk of HCC among non-HBV-infected population.

Carcinogenesis is a multistep process involving aberrations in a variety of cellular processes, such as DNA damage detection and repair [17]. As an important DNA damage-binding protein in the NER pathway, the alternation expression of DDB2 might affect the DNA repair capacity and thus contribute to cancer development, including HCC [18, 19]. Evidence has shown that DDB2 is downregulated in skin cancer, and the DDB2 knockout mice exhibited much higher incidence in developing UV-induced skin cancer [20]. Furthermore, the DDB2 deficient cells were resistant to p53-induced apoptosis in response to a variety of DNA-damaging agents by attenuating the barrier of p21^Waf1/Cip1^ [21]. It is shown that enhanced DDB2 expression protects mice from carcinogenic effects of chronic UV-B irradiation, and this protection effect is most likely mediated by accelerating the repair of photolesions [22]. Previous studies showed that polymorphisms in the *DDB2* gene may contribute to the etiology of lung cancer, gastric cancer, and head and neck cancer [23–25]. Our results suggested that *DDB2* rs1050244 polymorphism may play a role in the development of HCC.

SNPs located at miRNA-binding sites are likely to affect the expression of the miRNA target by influencing the binding ability of miRNA, thus modifying the individual susceptibility to cancer [26]. For example, Tan et al. [27] found that the CT/TT genotypes of SNP rs17592236 located in the 3′ UTR of FOXO1 decreased the risk of HCC when compared with the CC genotype. Further functional assays confirmed that SNP rs17592236 was a target site of miR-137 and affected the binding affinity of miR-137 to 3′ UTR in mRNA of FOXO1. Li et al. [28] found that SNP rs7963551 in the let-7 target site of *RAD52* 3′ UTR was significantly associated with HCC risk, and individuals who carried the SNP rs7963551 CC genotype had a decreased risk of HCC compared with those carried the AA genotype. They found that the AC/CC genotypes of rs7963551 were associated with increased expression of mRNA of *RAD52*. In our study, SNP rs1050244 is located in 3′ UTR of the *DDB2* gene and was predicted to be miRNA-binding sites (miR-133a and miR-197) (http://snpinfo.niehs.nih.gov/snpfunc.htm). It has been reported that the miR-133a acts as an antioncogene by inhibiting the expression of target genes, while miR-197 expression is upregulated in HCC, and miR-197 may regulate the expression of its downstream target genes at posttranscriptional level [29, 30]. In our study, we speculate that *DDB2* SNP rs1050244 may interfere with miRNA target interactions, leading to upregulation of DDB2 mRNA expression, thereby reducing the susceptibility to HCC. However, our speculation needs to be further confirmed by functional experiments.

Several limitations in our study should be considered. Firstly, this study is a hospital-based case-control study, and an uncontrolled selection bias exists. Secondly, the sample sizes in the subgroup analyses are relatively small, and thus the statistical power is limited. Lastly, functional assay is absent to uncover the underlying mechanism of the interaction effect between SNP rs1050244 and HBV infection on the risk of HCC in this study.

In conclusion, our study suggests that the *DDB2* rs1050244 C>T polymorphism was associated with the decreased risk of HCC among non-HBV-infected population. Further studies with larger sample sizes are needed to validate our findings.

## Figures and Tables

**Table 1:** Frequency distributions of selected variables in HCC cases and controls.

Variables	Cases *n* (%)	Controls *n* (%)	*P* ^a^
All subjects	1073	1119	
Age			0.172
≤48^b^	522 (48.6)	577 (51.6)	
>48	551 (51.4)	542 (48.4)	

Sex			0.338
Males	946 (89.5)	1001 (88.2)	
Females	127 (10.5)	118 (11.8)	

Smoking			<0.001
Never	625 (58.2)	866 (77.4)	
Ever	448 (41.8)	253 (22.6)	

Drinking			<0.001
Never	655 (61.0)	894 (79.9)	
Ever	418 (39.0)	225 (20.1)	

HBV infection			<0.001
(−)	174 (16.2)	1005 (89.8)	
(+)	899 (83.8)	114 (10.2)	

^a^Two-sided Chi-squared test. ^b^The median age of this population.

**Table 2:** Genotype frequencies of *DDB2* rs1050244 in cases and controls and their association with the risk of HCC.

Variants	Cases *n* (%)	Controls *n* (%)	*P* ^a^	Adj OR (95% CI)^b^	*P* ^b^
rs1050244					
CC	990 (92.26)	1006 (89.90)	0.153	1	
CT	80 (7.46)	109 (9.74)		0.77 (0.49–1.22)	0.270
TT	3 (0.28)	4 (0.36)		0.63 (0.07–6.15)	0.693
CT/TT	83 (7.74)	113 (10.10)	0.053	0.77 (0.49–1.21)	0.249

^a^Two-sided Chi-squared test for genotype distributions between cases and controls. ^b^Adjusted for age, sex, smoking status, drinking status, and HBV infection in logistic regression models.

**Table 3:** Stratified analysis for the associations between *DDB2* rs1050244 genotypes and HCC risk.

Variables	rs1050244 (cases/controls)		Adj OR (95% CI)	*P* ^a^	*P* ^b^
	CC	CT/TT	CC vs. CT/TT		
Age					0.195
≤48^c^	481/523	41/54	1.05 (0.52–2.14)	0.883	
>48	509/483	42/59	0.60 (0.33–1.11)	0.103	

Sex					0.928
Males	880/902	66/99	0.76 (0.46–1.26)	0.284	
Females	110/104	17/14	0.74 (0.23–2.45)	0.625	

Smoking					0.28
Never	566/780	59/86	0.91 (0.52–1.57)	0.728	
Ever	424/226	24/27	0.56 (0.24–1.27)	0.164	

Drinking					0.317
Never	597/802	58/92	0.88 (0.52–1.50)	0.646	
Ever	393/204	25/21	0.59 (0.24–1.47)	0.258	

HBV infection					**0.002**
(−)	168/896	6/109	**0.31 (0.13–0.72)**	**0.006**	
(+)	822/110	77/4	2.50 (0.89–7.00)	0.082	

^a^Adjusted for age, sex, smoking, drinking, and HBV infection in logistic regression models.  ^b^*P* value for interaction. ^c^The median age of this population.

## Data Availability

The data used to support the findings of this study are available from the corresponding author upon request.

## Supplementary Materials

Supplementary Materials Table 1S: the associations between DDB2 rs1050244 polymorphism and clinical features of HCC patients. .
