# Supplementary material for: Association between Single-Nucleotide Polymorphism in MicroRNA Target Site of DDB2 and Risk of Hepatocellular Carcinoma in a Southern Chinese Population
Source: Biomed Res Int. 2020 Feb 8;2020:8528747. doi: 10.1155/2020/8528747 (PMC7031712; doi:10.1155/2020/8528747)
Supplement: Supplementary Materials — Table 1S: the associations between DDB2 rs1050244 polymorphism and clinical features of HCC patients. . [file 8528747.f1.docx]

Table 1S. The associations between *DDB2* rs1050244 polymorphism and clinical features of HCC patients.

| variables | All Cases  (n=563) | rs1050244 (cases/controls) | | *P ^a^* |
| --- | --- | --- | --- | --- |
|  |  | CC | CT/TT |  |
| Tumor size (cm) |  |  | | 0.264 |
| <5 | 313 | 285 | 28 |  |
| ≥5 | 250 | 234 | 16 |  |
| Tumor number |  |  |  | 0.601 |
| Single | 452 | 418 | 34 |  |
| Multiple | 111 | 101 | 10 |  |
| BCLC stage |  |  |  | 0.391 |
| A/B | 366 | 340 | 26 |  |
| C/D | 197 | 179 | 18 |  |
| AFP level (ng/mL) |  |  |  | 0.144 |
| < 400 | 352 | 329 | 23 |  |
| ≥400 | 211 | 190 | 21 |  |
| Cancer embolus |  |  |  | 0.899 |
| No | 414 | 382 | 32 |  |
| Yes | 149 | 137 | 12 |  |
| Cirrhosis |  |  |  | 0.662 |
| No | 248 | 230 | 18 |  |
| Yes | 315 | 289 | 26 |  |

^a^ Two sides Chi-square test
